# The Open State Principle: a second-order framework for outcome interpretation and decision-making in aesthetic clinical systems

**DOI:** 10.3389/fmed.2026.1783056

**Published:** 2026-06-23

**Authors:** Andrea Felice Armenti

**Affiliations:** LA VISION Training Institute, Rome, Italy

**Keywords:** aesthetic outcomes, clinical decision-making, composite endpoints, inferential uncertainty, medical epistemology, observer dependence, outcome interpretation

## Abstract

Aesthetic clinical practice—including aesthetic surgery, non-surgical aesthetic medicine, dermatologic procedures, and biologically active interventions—has expanded substantially over the past two decades. This expansion has been accompanied by an increasing reliance on validated outcome instruments, patient-reported outcome measures (PROs), and composite endpoints for the evaluation of treatment success. Although these instruments demonstrate psychometric robustness and are widely accepted in clinical and regulatory contexts, they operate in the absence of a unifying theoretical framework that clarifies what aesthetic outcomes, naturalness, treatment response, and personalization epistemically represent. Current approaches commonly treat aesthetic outcomes as directly measurable properties of treated anatomy, while variability between observers, contexts, and instruments is frequently attributed to subjectivity or measurement noise. This perspective obscures the inferential structure underlying aesthetic evaluation and contributes to persistent conceptual fragmentation across surgical and non-surgical domains. In particular, it leaves unresolved the ontological status of outcome measures, the role of observer dependence, and the interpretability of composite scores aggregating heterogeneous sources of information. In this work, we introduce the Open State Principle (OSP), a second-order foundational framework that characterizes aesthetic clinical evaluation as operating on open states under irreducible biological, perceptual, and decision-level uncertainty. The OSP does not propose new metrics, invalidate existing validated scales, or prescribe clinical practice. Rather, it establishes minimal coherence constraints that any description of aesthetic outcome, naturalness, response to treatment, regeneration claims, or personalization must satisfy. From this principle, we derive necessary implications, including the impossibility of observer-independent aesthetic outcomes, the interpretation of treatment response as an inferential update relative to baseline expectations rather than a post-treatment state, the relational and context-dependent nature of naturalness judgments, the treatment of regeneration claims as causal hypotheses rather than observable endpoints, and the ontological fragility of composite scores that aggregate across distinct inferential levels. The OSP is fully compatible with existing validated instruments and regulatory practices. By reframing aesthetic evaluation as inference rather than direct measurement, it provides a unifying interpretive framework across aesthetic surgery and non-surgical aesthetic medicine, clarifies sources of non-replicability and apparent disagreement, and establishes a principled conceptual foundation for future methodological and mathematical developments in aesthetic clinical research.

## 1 Introduction

### 1.1 The expansion of aesthetic clinical practice

During the past two decades, aesthetic clinical practice has undergone substantial expansion, both in scope and in complexity. Contemporary aesthetic care encompasses a broad range of interventions, including aesthetic surgery, non-surgical aesthetic medicine, dermatologic procedures, energy-based technologies, and biologically active or so-called regenerative products. These approaches differ markedly in terms of mechanisms of action, invasiveness, reversibility, and temporal dynamics of effect, and contemporary practice patterns document a sustained increase in aesthetic procedural volume and diversification of procedural portfolios over time (1). In parallel, the maturation and diffusion of energy-based modalities in dermatologic and aesthetic settings has further expanded the intervention space (2). Biologically active interventions, including autologous platelet derivatives, have also proliferated within aesthetic and medical dermatology, contributing to additional heterogeneity in putative mechanisms and claims (3).

Despite this heterogeneity of means, aesthetic clinical practice is characterized by a striking homogeneity of evaluation questions. Across surgical and non-surgical domains, practitioners, researchers, regulators, and patients repeatedly converge on a limited set of concerns: whether an intervention was successful, whether the result appears natural, whether the outcome is satisfactory, and whether the treatment achieved its intended therapeutic goal. This convergence is consistent with the longstanding recognition that, in appearance-altering disciplines, evaluation is frequently organized around patient-centered constructs and interpretive judgments rather than around a single objective endpoint (4).

This convergence suggests that, beneath the diversity of procedures, aesthetic clinical practice addresses a shared problem space. The same concepts—outcome, response, naturalness, improvement, and personalization—are invoked to evaluate interventions that range from minimally invasive injectable treatments to irreversible surgical transformations. However, these concepts are rarely examined as such and are typically assumed to be self-evident or directly observable. The resulting reliance on implicit construct assumptions is reinforced by the practical necessity of communicating the results between observers and settings, even when the inferential status of these constructs is not explicitly specified (4).

### 1.2 Proliferation of outcomes without a unifying theory

In parallel with the expansion of aesthetic practice, there has been a proliferation of instruments designed to evaluate aesthetic outcomes. These include validated rating scales, patient-reported outcome measures (PROs), structured clinical assessments, photographic evaluations, and composite endpoints that combine multiple dimensions of change. Systematic reviews in facial plastic surgery have documented the rapid growth and heterogeneity of outcome instruments, including patient-reported, observer-reported, and objective measures (5). Many contemporary PRO instruments in aesthetic contexts have demonstrated strong psychometric performance, including reliability, validity, and responsiveness, and have been positioned as central components of outcome evaluation in plastic surgery (4, 6).

However, the coexistence of multiple outcome measures has not been accompanied by a unifying theoretical account of what these measures represent. Identical terms–such as “improvement,” “response,” or “natural appearance”–are often operationalized differently across instruments, studies, and clinical contexts. As a result, outcomes that appear comparable at a linguistic level may in fact refer to distinct constructs, inferred at different levels of description, even when each instrument is locally valid within its intended scope (5, 6).

This situation gives rise to incommensurability between studies and persistent difficulties in interpretation and comparison. Variability between observers, instruments, and settings is often attributed to subjectivity or measurement noise, rather than being examined as a structural feature of the aesthetic evaluation itself. The widespread use of composite endpoints further accentuates these interpretive challenges: composite measures can summarize complex multidimensional information, but their validity and meaning depend on explicit measurement-theoretic assumptions that are often left implicit (7). Consequently, the conceptual basis of aesthetic results remains largely implicit, even as their instrumental use becomes increasingly standardized (4, 7).

### 1.3 Lessons from other domains before foundational principles

Similar patterns of conceptual fragmentation have been observed in other scientific domains prior to the introduction of foundational principles. In neurosciences, for example, diverse experimental paradigms, behavioral measures, and clinical assessments have been interpreted through multiple partially overlapping frameworks, motivating efforts to articulate unifying principles capable of constraining interpretation across heterogeneous data sources (8).

In such cases, progress was not achieved by replacing existing instruments or invalidating established measures. Rather, it emerged through the articulation of principles that clarified the conditions under which diverse observations could be understood as instances of a common underlying structure. These principles operated at a level distinct from the empirical description, imposing coherence constraints on how phenomena could be meaningfully related, interpreted, and compared (8).

The relevance of these precedents for aesthetic clinical practice lies not in the specifics of their formalization, but in the recognition that conceptual unification often precedes, and enables, methodological refinement. A second-order framework can therefore function as an interpretive constraint system: it does not compete with instruments at the measurement level, but constrains the conditions under which instrument outputs can be coherently interpreted and compared (9).

### 1.4 Aim and scope of the present work

The purpose of the present work is to introduce a foundational principle of inference for esthetic clinical practice. This principle is intended to clarify the epistemic status of core aesthetic concepts such as outcome, naturalness, response to treatment, regeneration, and personalization by situating them within a unified inferential framework. Throughout this work, “open state” denotes a state defined at the level of description and decision-making, not a complete or observer-independent representation of underlying reality.

Importantly, this work does not propose new outcome measures, does not invalidate existing validated scales or PROs, and does not offer clinical guidelines or normative prescriptions. Its scope is deliberately limited to the formulation of minimal coherence constraints that any description of aesthetic evaluation must satisfy, regardless of the specific intervention, instrument, or clinical context involved.

By reframing aesthetic clinical practice in inferential terms, the proposed framework seeks to provide a common conceptual ground across aesthetic surgery, non-surgical aesthetic medicine, and related domains, while remaining fully compatible with current clinical and regulatory practices, including established approaches to PRO development, validation, and use (4, 6). The contribution of the OSP is therefore not empirical extension but conceptual constraint: it delineates the conditions under which existing evidence, instruments, and decisions can be coherently interpreted. In the absence of such coherence constraints, aesthetic outcomes cannot be coherently defined as observer-independent evaluative quantities.

## 2 Defining aesthetic clinical systems

### 2.1 From anatomy and procedures to systems

Aesthetic clinical practice is traditionally described in procedural terms. Interventions are commonly classified according to anatomical targets, surgical or non-surgical techniques, materials, devices, or energy sources, and outcomes are frequently discussed as properties of treated structures. This procedure-centered description is effective for operative planning, technical communication, and device or product characterization. However, it becomes insufficient when addressing evaluative concepts such as outcome quality, naturalness, or treatment success, which extend beyond anatomical description alone (4).

A procedure-centered perspective implicitly assumes a relatively direct mapping between anatomical modification and aesthetic outcome. Under this assumption, changes in the characteristics of the form, volume, or tissue are expected to correspond in a stable manner to perceived improvement or success. The empirical experience in aesthetic practice challenges this assumption. Identical anatomical changes can lead to divergent aesthetic evaluations between observers, contexts, and time points, while markedly different procedures can produce outcomes perceived as equivalent or comparable (5). These observations indicate that aesthetic evaluation cannot be reduced to procedural description or anatomical alteration alone.

To address this limitation, the present framework introduces the aesthetic clinical system as the appropriate unit of analysis. An aesthetic clinical system is not defined by a specific intervention, but by the interaction of multiple components that jointly determine aesthetic evaluation and decision-making. This system-oriented perspective aligns with broader approaches in clinical research that emphasize the role of context, observation, and interpretation in outcome evaluation rather than treating outcomes as direct readouts of intervention effects (10).

Specifically, an aesthetic clinical system comprises:

- a latent biological state, encompassing anatomical, structural, and physiological properties that are not directly observable as aesthetic qualities;
- a set of observations, including clinical examination, imaging, photographic documentation, and perceptual impressions;
- one or more observers (human or algorithmic), such as clinicians, patients, third-party assessors, or automated systems, each operating with implicit or explicit perceptual and evaluative models;
- therapeutic decisions, including the selection, timing, and modification of interventions under conditions of uncertainty.

Within this system, aesthetic outcomes do not emerge as direct consequences of procedures, but as inferential products arising from the interaction between latent states, available observations, and observer models. This system-level perspective applies uniformly across aesthetic surgery, non-surgical aesthetic medicine, dermatology, and related domains, regardless of differences in technical execution, invasiveness, or biological mechanism (4, 5).

### 2.2 Levels of description in aesthetic clinical systems

Aesthetic clinical systems operate simultaneously across multiple levels of description. The failure to distinguish these levels contributes substantially to conceptual ambiguity in the assessment and interpretation of the results. Therefore, the present framework introduces a formal distinction between four primary levels of description, consistent with established distinctions in the research of health outcomes and clinical decision-making (10).

The biological–structural level refers to changes in anatomy, tissue composition, biomechanics, and physiology induced by intervention or natural processes. These changes constitute the substrate on which the aesthetic evaluation is based, but they are not themselves aesthetic outcomes.

The perceptual level concerns how biological states give rise to visual, tactile, or otherwise perceptible features. This level captures properties such as contour, symmetry, texture, and proportion as they are perceived, rather than as they are anatomically instantiated.

The subjective level encompasses individual judgments, preferences, expectations, and satisfaction. This includes patient-reported results and self-assessments, which reflect personal interpretations of perceptual changes rather than direct measurements of biological states (11).

The decision level involves clinical and regulatory judgments, including treatment planning, response classification, and determination of success or failure. At this level, information from other levels is integrated to support action under uncertainty, in line with the decision-oriented use of clinical outcome data (12).

These levels are conceptually distinct but operationally intertwined. Aesthetic instruments and outcome measures frequently aggregate information across multiple levels without explicitly acknowledging their heterogeneity. Although such aggregation may be pragmatically useful, it obscures the inferential structure of aesthetic evaluation and complicates the comparison between studies and contexts (7).

By explicitly distinguishing levels of description, the aesthetic clinical system framework prepares the ground for a principled analysis of outcome measures, scale construction, and decision-making processes. This distinction also anticipates the inferential constraints formalized by the OSP introduced in the following section.

For completeness, a minimal and deliberately underspecified formal skeleton of the Aesthetic Inference Principle is provided as Supplementary material S1, with the sole purpose of illustrating its inferential structure. Additional Supplementary material that address formal results and terminological fixation are introduced later in the manuscript, where their relevance becomes explicit.

## 3 The Open State Principle (OSP)

### 3.1 Formal statement of the principle

The Open State Principle (OSP) states that aesthetic clinical evaluation must be understood as operating on open states: states that cannot be fully specified, closed, or directly observed, but only inferred through partial observations and observer-dependent models. An open state is “open” with respect to any attempt at observer-independent closure: it is not fully specifiable by measurement alone because it is defined relationally across inferential levels. This characterization is consistent with established accounts of latent-state inference in complex clinical and perceptual domains, where the target of evaluation cannot be exhaustively captured by only observable variables (9).

Within this framework, aesthetic outcomes, naturalness, response to treatment, and personalization are not directly observed properties of treated anatomy, but inferential constructs that emerge from the interaction between latent biological states, available observations, observer-dependent models, and decision processes. This inferential perspective aligns with broader epistemological approaches in medicine and cognitive science that treat outcomes as beliefs updated conditioned on evidence rather than as direct readouts of the underlying reality (10). While the OSP is compatible with Bayesian and decision-theoretic frameworks, it is not derived from them and does not presuppose any specific formal implementation.

The novelty of the OSP does not lie in the introduction of new empirical variables but in the explicit formulation of coherence constraints that are currently treated implicitly and inconsistently across the field. In this sense, the OSP functions as a coherence principle for aesthetic clinical evaluation rather than as a competing measurement proposal. Similar second-order principles have been shown to play a clarifying role in other domains by constraining interpretation without prescribing specific empirical models (8).

The OSP does not describe specific biological mechanisms, procedural effects, or perceptual pathways. Rather, it imposes a set of minimal coherence constraints on how aesthetic phenomena can be meaningfully described, compared, and interpreted. Any account of aesthetic clinical outcomes that violates these constraints is not empirically false but conceptually incomplete, insofar as it conflates inferential levels or treats relational constructs as directly observable quantities (9).

As a second order principle, the OSP does not compete with existing outcome measures, clinical instruments, or regulatory frameworks. Instead, it specifies the conditions under which such instruments can be coherently situated within a unified conceptual structure, preserving their practical utility while clarifying their epistemic scope (4).

### 3.2 Inferential assumptions

The OSP is based on a small number of inferential assumptions that are implicit in aesthetic clinical practice, but rarely made explicit.

First, aesthetic evaluation presupposes the existence of latent aesthetic states. Properties such as harmony, attractiveness, or natural appearance are not directly accessible to measurement but are inferred from observable proxies. Even when anatomical changes are precisely quantified, their aesthetic significance remains mediated by interpretation. This reliance on latent constructs is a defining feature of outcome assessment in domains where evaluative judgments cannot be reduced to single observable variables (10).

Second, aesthetic inference relies on observations that are inherently noisy and incomplete. Clinical examination, imaging, photography, and subjective impressions provide partial and context-dependent information about the underlying states. No increase in observational resolution can fully eliminate this uncertainty, as aesthetic judgments depend on interpretation rather than direct detection. This form of irreducible uncertainty has been extensively discussed in the context of observer-dependent assessment and measurement theory (9).

Third, aesthetic decision-making operates under irreducible uncertainty at multiple levels. Biological responses to the intervention vary between individuals and over time. Perceptual evaluations vary between observers and contexts. Clinical decisions must therefore be made without access to a fully specified or deterministic mapping between intervention and outcome. Decision-making under such conditions is described more appropriately within an inferential and decision-theoretic framework than within a deterministic or purely descriptive model (12).

Together, these assumptions indicate that aesthetic clinical practice is not adequately described by deterministic mappings from intervention to outcome. Instead, it is more appropriately characterized as an inferential process, in which beliefs about latent states are continuously updated in light of new observations and constrained by decision-level considerations (8). Importantly, increases in observational resolution – including advanced three-dimensional imaging, automated morphometric analysis, or AI-based assessment pipelines – do not alter the inferential status of aesthetic outcomes. Such technologies refine the space of observations, but do not eliminate observer-indexed interpretation, baseline expectations, or decision-level criteria.

### 3.3 Breadth of the domain

The OSP is applied uniformly throughout the entire spectrum of aesthetic clinical practice. Aesthetic surgery, non-surgical aesthetic medicine, dermatologic procedures, and technology-based interventions represent different means of acting upon biological substrates, but they do not differ in their epistemic status.

From the perspective of the OSP, these domains constitute instances of the same inferential problem: how to act upon a latent biological state in order to produce observations that will be interpreted as desirable under observer-dependent evaluative models. Differences in invasiveness, reversibility, or mechanism of action alter the dynamics of inference, but not its fundamental structure. This interpretation is consistent with system-oriented approaches to clinical evaluation that emphasize inference over procedure-specific categorization (4).

Surgical interventions, due to their irreversibility and large effect sizes, serve as a limiting case that makes the inferential character of the outcome assessment maximally explicit rather than exceptional. In such contexts, the dependence of outcome interpretation on baseline expectations, observer models, and decision thresholds becomes particularly evident.

This system-level perspective is especially important for concepts such as regeneration. Within the OSP framework, “regeneration” does not define a privileged domain of aesthetic practice. Rather, it represents a causal hypothesis about the biological processes that underlie the observed changes. As such, it cannot serve as a foundational category for the evaluation of the result, but must be evaluated inferentially alongside alternative explanations, regardless of whether the intervention is surgical, injectable, technological, or biological (7).

By treating all aesthetic interventions as epistemically equivalent instances of inference under uncertainty, the OSP provides a unified conceptual foundation capable of encompassing the full breadth of aesthetic clinical systems.

## 4 Axioms of the Open State Principle

### 4.1 Latent aesthetic states

Aesthetic properties relevant to clinical evaluation—such as harmony, attractiveness, balance, or natural appearance—do not constitute directly observable variables. Rather, they correspond to latent aesthetic states that cannot be accessed independently of interpretation. This characterization is consistent with established distinctions in outcome research between observable indicators and the latent constructs they are intended to represent (10).

Observable quantities, including anatomical measurements, imaging data, photographic representations, and clinical findings, provide indirect and partial information about these states. Even when such observations are precise, reproducible, or instrumentally validated, their aesthetic significance is not inherent to the observation itself. Instead, it emerges through an inferential interpretation conditioned on contextual assumptions and evaluative models (9).

Consequently, aesthetic clinical evaluation cannot be reduced to the measurement of surface features or structural changes alone. It necessarily involves inference about latent states that remain undetermined by any finite set of observations. This underdetermination is not a methodological deficiency, but a defining characteristic of evaluative constructs that lack one-to-one correspondence with observable variables (12).

### 4.2 Observer-dependence and relational judgment

All aesthetic judgments are inherently relational, arising from the interaction between a latent aesthetic state and an observer endowed with a perceptual and evaluative model. No aesthetic judgment can be defined independently of the observer who makes it, because the act of evaluation presupposes a perceptual, cognitive, and contextual framework (9).

Observers may include clinicians, patients, or third parties, each operating under different expectations, previous experiences, cultural norms, and contextual constraints. As a result, identical observations may lead to divergent aesthetic evaluations without implying error, bias, or inconsistency. This divergence reflects differences in observer models rather than measurement failures (4).

Observer dependency is therefore not a secondary source of noise to be corrected or eliminated, but a constitutive feature of aesthetic inference. Any framework that treats aesthetic outcomes as observer-independent properties of treated anatomy is, by construction, incomplete, insofar as it neglects the relational conditions under which aesthetic judgments acquire meaning (9).

### 4.3 Irreducible uncertainty

Aesthetic clinical systems operate under irreducible uncertainty at multiple levels. Biological responses to intervention are variable between individuals and over time and are only partially predictable even under controlled conditions. Perceptual interpretations are context-sensitive and observer-dependent, and decision-making must integrate incomplete, heterogeneous, and sometimes conflicting sources of information (10).

This uncertainty cannot be eliminated through increased measurement precision, expanded data collection, or stricter standardization alone. Although such measures may reduce specific sources of variability, they cannot resolve the fundamental indeterminacy associated with latent states and relational judgment. Similar limits have been described in other domains of clinical and statistical inference, where uncertainty reflects the structural features of the problem rather than the remediable error (9).

Consequently, aesthetic clinical practice cannot be adequately modeled as a deterministic mapping from intervention to outcome. Uncertainty is not an implementation limitation, but a structural property of the domain, requiring an inferential rather than purely descriptive account of evaluation and decision-making (8).

### 4.4 Outcomes as inferential updates

Within the OSP framework, an aesthetic outcome is not defined as a post-intervention state of the system. Rather, it corresponds to an inferential update relative to an expected or anticipated state. This interpretation aligns with broader conceptions of outcomes in health research as changes in belief or assessment conditioned on new evidence rather than as absolute post-treatment measurements (10).

Baseline assessments implicitly encode prior expectations regarding aesthetic appearance, treatment effect, and acceptability. Post-intervention observations provide new evidence that modifies these expectations. The outcome of treatment is therefore expressed as a change in belief in the latent aesthetic state, rather than as an absolute value attributed solely to the intervention (12).

This interpretation applies uniformly across the outcome measures, including scales rated by clinicians, patient-reported outcomes, and composite endpoints. In all cases, the reported result reflects an update in inference conditioned on observation and prior expectations, not a direct measurement of aesthetic quality as an observer-independent quantity (4).

### 4.5 Coherence across inferential levels

The aesthetic clinical evaluation integrates information across multiple levels of description, including representations at the biological, perceptual, subjective, and decision-level. These levels are conceptually distinct and cannot be coherently combined without explicit justification. Treating variables from different inferential levels as if they were commensurable obscures the structure of inference underlying outcome evaluation (7).

It is not epistemically coherent to aggregate variables belonging to different inferential levels unless a mapping between those levels is specified. Composite scores that combine heterogeneous components may serve practical decision-making purposes but do not correspond to natural or observer-independent quantities. Therefore, your interpretation is dependent on the assumptions embedded in your construction (7).

The requirement of coherence across inferential levels does not prohibit aggregation or simplification. Rather, it constrains how such operations can be meaningfully interpreted and compared across contexts. Failure to respect this constraint leads to ambiguity in outcome interpretation and limits the generalizability of findings across studies and settings.

## 5 Necessary implications and derivations

The implications presented in this section necessarily follow from the axioms of the OSP. They do not constitute additional assumptions, empirical hypotheses, or normative claims. Rather, they delineate the conceptual consequences that any coherent account of aesthetic clinical evaluation must satisfy once aesthetic outcomes are treated as inferential constructs operating on latent states under uncertainty.

### 5.1 Impossibility of observer-independent outcomes

Given that aesthetic states are latent (Axiom 4.1) and that aesthetic judgments are inherently relational (Axiom 4.2), it follows that observer-independent aesthetic outcomes cannot exist. An outcome cannot be defined as a property of treated anatomy alone, nor as an invariant feature of an intervention. This conclusion is consistent with broader epistemological analyses of evaluative constructs, which emphasize that judgments lacking observer specification are underdetermined by observable data alone (9).

Different observers, such as clinicians, patients, or third-party assessors, may legitimately infer different aesthetic outcomes from the same set of observations, without the fact that any of these inferences are incorrect. This variability does not indicate measurement failure, but reflects the constitutive role of observer models in aesthetic evaluation, as documented in patient-reported and observer-reported outcome research (4).

Consequently, attempts to define universal or context-free aesthetic outcomes implicitly rely on unacknowledged assumptions about observer alignment. In the absence of explicit model alignment, outcome variability must be regarded as a structural feature of aesthetic clinical systems rather than as noise to be eliminated (9).

#### 5.1.1 Ill-posedness of aesthetic outcome interpretation without inferential specification

If aesthetic outcomes are treated as observer-independent functions of post-intervention observations alone, then outcome interpretation is not merely incomplete, but formally ill-posed. In an aesthetic clinical system, evaluative outputs arise from inferential updates over latent states and are constitutively dependent on observer-indexed models and baseline expectations. Consequently, for the same observation y∈Y, different observers $o_{1},o_{2}\in O$ may legitimately produce distinct inferential updates. Under these conditions, no observer-invariant mapping g:Y→Z can, in general, represent “the” aesthetic outcome. The absence of such a mapping implies that observer-independent outcomes are not empirically false but are underdetermined and, therefore, undefined as coherent quantities. The OSP makes this implicit ill-posedness explicit by specifying the inferential indexing required for aesthetic outcome evaluation to be meaningfully defined.

#### 5.1.2 Proposition (non-existence of observer-invariant aesthetic outcomes)

Consider an aesthetic clinical system in which outcomes are defined as inferential updates over latent states, conditioned on observer-indexed models and baseline expectations. If there exist two observers $o_{1},o_{2}\in O$ such that, for the same observation y∈Y, the corresponding inferential updates differ, then no observer-invariant mapping g:Y→Z can, in general, represent “the” aesthetic outcome.

#### 5.1.3 Proof sketch

Assume, by contradiction, that there exists an observer-invariant outcome mapping *g*(*y*). By definition, *g*(*y*) must coincide with the evaluative output produced by any observer given the same observation. However, if outcome evaluation depends constitutively on observer-indexed inferential updates, then for some *y* there exist *o*_1_, *o*_2_ such that the corresponding updates differ. In this case, no single mapping *g*(*y*) can satisfy both evaluations simultaneously. Therefore, in general, an observer-invariant esthetic outcome mapping cannot exist.

The above result does not depend on any specific probabilistic formalism or implementation choice. Rather, it follows from minimal assumptions regarding latent states, observer dependence, and inferential updating. A more explicit formal treatment, including precise definitions and additional propositions, is provided in the Supplementary material S4, with the sole purpose of demonstrating that these coherence constraints admit a rigorous mathematical articulation.

### 5.2 Treatment response as inferential transformation

Within the OSP framework, the response to treatment cannot be identified with the post-intervention state of the system. Since the results are defined as inferential updates (Axiom 4.4), the response to treatment corresponds to a transformation in belief in a latent esthetic state, conditioned on new observations. This interpretation is consistent with the outcome theory in health research, where change is defined relative to baseline expectations rather than as an absolute post-treatment quantity (10).

Baseline assessments encode previous expectations about appearance, effect magnitude, and acceptability. Post-treatment observations provide evidence that modifies these expectations. The response is therefore relational and comparative, defined relative to an anticipated state and not as an absolute quantity attributable solely to the intervention (12).

This interpretation applies uniformly across clinical contexts, including both surgical and non-surgical interventions. Classifications such as “responder” and “non-responder,” when used without explicit reference to baseline expectations and inferential criteria, are conceptually underdetermined and risk conflating descriptive change with evaluative judgment (4).

### 5.3 Limits of naturalness as an absolute scale

Naturalness is frequently treated as a scalar attribute of aesthetic outcomes. However, given the observer-dependent and relational nature of aesthetic judgment (Axiom 4.2), naturalness cannot be defined as an absolute or observer-independent quantity. Evaluative research in the clinical and perceptual domains has consistently shown that naturalness judgments are based on contextual framing and observer expectations rather than on anatomical configuration alone (9).

Perceptions of naturalness depend on contextual norms, cultural expectations, temporal framing, and the previous exposure of the observer to aesthetic interventions. The same anatomical configuration may be perceived as natural or artificial depending on these factors, even when observations are identical.

As a result, any scale purporting to measure naturalness is necessarily local in scope. Such scales may be valid within specific contexts and observer populations, but cannot be assumed to provide a universal metric. Comparisons across studies or settings therefore require explicit alignment of perceptual and evaluative models to avoid category errors in interpretation (4).

### 5.4 Regeneration as a causal narrative, not an outcome

Claims labeled as “regeneration” in aesthetic clinical practice refer to inferred biological processes underlying observed changes. Under the OSP, regeneration corresponds to a causal narrative, rather than to an observable outcome. Observed improvements, such as changes in texture, thickness, elasticity, or appearance, do not demonstrate regeneration in themselves, as they are compatible with multiple explanatory hypotheses.

Such observations may reflect structural modification, redistribution, compensatory processes, or perceptual effects, among other possibilities. Without explicit causal modeling and differentiation among competing explanations, regeneration remains an inferential claim rather than an endpoint that can be directly measured (7).

Consequently, regeneration cannot serve as a foundational category for the evaluation of results. Instead, it must be treated as a hypothesis about the underlying processes, evaluated inferentially alongside alternative explanations, regardless of whether the intervention is biologically active, minimally invasive, technological, or surgical.

### 5.5 Ontological fragility of composite scores

Composite scores that aggregate multiple components are widely used in aesthetic clinical research. However, given the requirement of coherence between inferential levels (Axiom 4.5), such scores lack a stable ontological status unless their internal mappings are explicitly defined. Measurement-theoretic analyses have emphasized that composite endpoints derive their meaning from modeling assumptions rather than direct correspondence to a single underlying construct (7).

Components of composite instruments often span biological, perceptual, subjective, and decision-level variables. Aggregating these heterogeneous elements without an explicit inferential model produces quantities whose interpretation is context-dependent and non-generalizable.

This does not render the composite scores invalid or useless. Rather, it constrains their meaning: they function as local decision-support tools rather than as natural measures of aesthetic states. Treating them as universally comparable results exceeds their inferential scope and risks misinterpretation between studies and settings (7).

### 5.6 Personalization as decision-making under uncertainty

Personalization is frequently invoked as a defining feature of aesthetic clinical practice. Within the OSP framework, personalization cannot be understood as deterministic optimization but must be interpreted as decision-making under uncertainty (Axiom 4.3). This view aligns with established accounts of clinical decision-making, in which actions are selected under incomplete information and probabilistic expectations (10).

Clinical decisions are made in the presence of incomplete information on biological response, perceptual interpretation, and patient preference. Personalization therefore reflects the selection of an action based on expected consequences, rather than the identification of a uniquely correct intervention.

This interpretation applies to the full range of aesthetic clinical systems. In surgical contexts, where interventions are often irreversible and the effects are long-lasting, uncertainty is amplified rather than reduced. Thus, surgery represents a limiting case that highlights the inferential nature of personalization, rather than an exception to it.

## 6 Relation to validated scales, PROs, and regulatory practice

### 6.1 What validation guarantees—and what it does not

Validated scales and PROs play a central role in aesthetic clinical research and practice. Psychometric validation establishes important properties, including reliability, internal consistency, sensitivity to change, and construct validity within a specified context. Contemporary standards for the development and evaluation of outcome measures, such as the COSMIN framework, further formalize these psychometric requirements without addressing the fundamental epistemic status of the constructs being measured (13). These properties justify the use of such instruments for comparison, monitoring and decision-making in both clinical trials and routine practice (11, 12).

However, validation addresses instrument performance rather than the foundational status of the constructs being measured. Psychometric robustness does not determine, by itself, whether an outcome corresponds to a directly observable quantity, a latent variable, or an inferential construct. Nor does it specify the inferential level—biological, perceptual, subjective, or decision–level—at which a given measure operates. This distinction is well recognized in outcome research, where validated instruments may target heterogeneous constructs despite comparable measurement properties (10).

This distinction should not be interpreted as a limitation of validation procedures, but as a clarification of their scope. Validation ensures that an instrument behaves consistently and meaningfully relative to its intended use; it does not provide a theory of what aesthetic outcomes fundamentally are. Conflating psychometric validity with epistemic completeness risks obscuring the inferential structure underlying aesthetic evaluation and may encourage overinterpretation of instrument output beyond their intended domain (12).

### 6.2 Interpreting existing instruments under the OSP

Within the OSP framework, existing validated instruments – including clinician-rated scales, wrinkle severity scales, and PROs such as FACE–Q -are interpreted as local decision-support tools and inferential proxies rather than as direct measures of latent aesthetic states. This interpretation is consistent with established views in plastic surgery and outcomes research, which emphasize that PROs and observer-rated scales capture specific perspectives rather than exhaustive representations of treatment effects (4, 6).

These instruments operationalize particular viewpoints on aesthetic evaluation, often corresponding to specific observers, contexts, and decision criteria. Their outputs therefore reflect inferential updates conditioned on predefined assumptions, framing, and observer models, rather than observer-independent properties of treated anatomy. The divergence between instruments should therefore be understood as reflecting differences in inferential perspective rather than as evidence of instrument failure (4).

Importantly, this interpretation does not diminish the clinical or scientific value of validated instruments. In contrast, it situates them coherently within a broader inferential structure. Instruments that are explicitly recognized as local and context-dependent can be used more transparently, compared more carefully, and interpreted more accurately across studies and clinical settings (6).

The OSP is therefore fully compatible with the continued use of validated scales and PROs. It neither challenges their legitimacy nor proposes their replacement. Instead, it provides a conceptual framework for understanding why different instruments may yield divergent but equally valid assessments and why aggregation across instruments requires explicit justification at the level of inference rather than reliance on psychometric performance alone.

### 6.3 Regulatory acceptance and inferential interpretation

Regulatory frameworks governing aesthetic clinical research, including those associated with clinical trials and approvals by agencies such as the U.S. Food and Drug Administration (FDA) and the European Medicines Agency (EMA), prioritize consistency, reproducibility, and decision relevance. Validated outcome measures and PROs satisfy these requirements by providing standardized tools to evaluate treatment effects and support regulatory decision-making (11).

The OSP does not conflict with regulatory practice, because it operates at a different level of abstraction. Regulatory sufficiency concerns whether the available evidence supports safe and effective use under specified conditions. Epistemic completeness refers to how aesthetic outcomes are conceptually defined and interpreted. These questions are complementary rather than competing.

Recognizing this distinction allows regulatory decisions to be understood as pragmatic determinations under uncertainty, rather than as endorsements of a particular ontological interpretation of aesthetic outcomes. From the OSP perspective, regulatory acceptance reflects the adequacy of an inferential framework for decision-making within defined constraints, not the existence of observer-independent aesthetic quantities (10).

By maintaining a clear separation between regulatory sufficiency and foundational theory, the OSP preserves full compatibility with existing approval processes while clarifying their conceptual limits. This separation enables aesthetic clinical research to advance methodologically and interpretively without conflating practical decision criteria with claims of theoretical completeness.

## 7 Mathematical formalization: limits and future directions

### 7.1 Why full formalization is premature

Although the OSP is structurally compatible with formal inferential frameworks, a complete mathematical formalization of aesthetic clinical systems is currently premature. This limitation does not arise from a lack of available mathematical tools, but from the present epistemic and empirical state of the domain.

First, aesthetic clinical practice lacks standardized generative models that specify how latent aesthetic states give rise to observable data across different interventions, modalities, and contexts. In contrast to domains in which the relationship between latent states and observations is relatively stable and well-characterized, aesthetic clinical systems involve heterogeneous biological substrates, diverse observational modalities, and multiple observer-dependent evaluative models. Under such conditions, specifying a single generative model would require assumptions that exceed available empirical constraints and risk embedding unexamined normative commitments (8).

Second, the domain is intrinsically heterogeneous. Surgical and non-surgical interventions differ markedly in reversibility, temporal dynamics, and causal structure. Patient-reported outcomes, clinician ratings, and perceptual assessments operate at different inferential levels and are governed by distinct sources of uncertainty. Forcing a single fully specified mathematical model at this stage would risk conflating fundamentally different forms of uncertainty and obscuring, rather than clarifying, the inferential structure of aesthetic clinical evaluation (9).

For these reasons, the present work deliberately refrains from proposing a complete formal implementation of the OSP. This restraint is consistent with methodological analyzes that emphasize that premature formalization can hinder conceptual clarity when foundational distinctions have not yet stabilized (14).

### 7.2 Inferential structure without heavy mathematics

Despite the absence of full formalization, the inferential structure implied by the OSP can be articulated without recourse to heavy mathematics. Aesthetic clinical systems can be consistently described in terms of latent states, observations, belief updates, and decisions under uncertainty, without specifying concrete probability distributions, likelihood functions, or optimization criteria.

This abstract structure is isomorphic to well-established inferential frameworks, including Bayesian inference and decision theory, which provide a general language for reasoning under uncertainty without prescribing domain-specific models (15). The purpose of this abstraction is not to operationalize aesthetic evaluation or to propose computable models, but to ensure conceptual coherence across descriptions of outcome, treatment response, naturalness, and personalization.

By maintaining this level of abstraction, the OSP remains applicable across the full breadth of aesthetic clinical systems while avoiding premature formal commitments that the field is not yet equipped to support. The emphasis is therefore placed on structural compatibility rather than on mathematical completeness.

### 7.3 Roadmap for future formal developments

The OSP defines a programmatic direction for future mathematical developments rather than a finished formal theory. Several avenues of formalization naturally follow from the principle once sufficient empirical, conceptual, and methodological constraints become available.

Future work may include:

- specification of state spaces representing latent aesthetic configurations at appropriate biological and perceptual resolutions;
- the development of observation models linking latent states to clinical measurements, perceptual assessments, and patient-reported data;
- the formalization of cost or utility functions that encode clinical priorities, aesthetic preferences, and patient expectations.

Such developments are intended as domain-specific extensions of the OSP rather than as prerequisites for its validity. The principle itself imposes coherence constraints on any such formalization, without prescribing a unique mathematical implementation or privileging a specific inferential formalism.

A minimal mathematical articulation of the inferential structure implied by the OSP is provided in Supplementary material S2. Formal propositions and proof sketches supporting the necessity claims advanced in this section, together with an explicit inferential glossary fixing terminology, are provided in Supplementary materials S3, S4.

## 8 Implications for research design and interpretation

The OSP has direct implications for how aesthetic clinical research is designed, interpreted, and compared across contexts. These implications do not require specific methodologies or invalidate existing approaches. Rather, they clarify the inferential commitments underlying common research practices and explain why certain forms of variability, limited reproducibility, and apparent disagreement are to be expected when outcomes are treated as inferential constructs operating under uncertainty.

### 8.1 Implications for study design

From the perspective of the OSP, study design in aesthetic clinical research necessarily involves the specification –explicit or implicit—of inferential assumptions. Baseline assessments encode prior expectations regarding latent aesthetic states, while outcome measures operationalize specific observational and evaluative models. This structure is consistent with established outcomes research analyzes, which emphasize that endpoints do not simply record change, but formalize particular questions about change (10).

Recognizing this structure encourages greater transparency in the selection and interpretation of outcome measures. Studies that clearly specify the observer perspective, contextual assumptions, and evaluative criteria underlying their endpoints are better positioned to produce interpretable and comparable results. In contrast, designs that conflate multiple inferential levels without explicit justification risk generating outcomes that are difficult to contextualize beyond their immediate setting (12).

The OSP does not prescribe which outcomes should be used. Instead, it highlights the importance of aligning the study design with the inferential level at which conclusions are intended to be drawn. Failure to maintain this alignment can lead to ambiguity in interpretation, even when the instruments are psychometrically robust.

These considerations have direct consequences for endpoint selection, stratification strategies, and the interpretation of effect heterogeneity in clinical trials. The apparent heterogeneity may reflect differences in inferential framing rather than an underlying inconsistency of treatment effects (4).

### 8.2 Comparing surgical and non-surgical trials

Comparisons between surgical and non-surgical aesthetic interventions are often challenging due to differences in invasiveness, reversibility, and temporal dynamics. These differences are often treated as methodological obstacles or sources of bias in comparative research. Methodological frameworks for non-pharmacological and surgical trials explicitly recognize these structural differences, emphasizing that variation in trial design reflects differences in intervention complexity rather than inferiority of evidence (16).

Within the OSP framework, such comparisons are understood as involving distinct inferential contexts rather than fundamentally different epistemic domains. Surgical and non-surgical trials operate on the same latent aesthetic states but differ in the nature, persistence, and interpretability of the evidence they generate. This distinction mirrors broader discussions in clinical research on the comparability of interventions with differing causal structures and temporal profiles (10).

This perspective explains why outcomes from surgical and non-surgical studies may not be directly comparable, even when similar instruments are used. Differences in observed effects reflect differences in inferential structure, baseline expectations, and decision thresholds, rather than necessarily indicating inconsistency or methodological failure. Explicit recognition of these differences allows comparisons to be framed with more caution and interpreted more coherently (4).

### 8.3 Imaging adjuncts and bedside diagnostics within the inferential framework

A relevant practical extension of these considerations concerns the increasing use of high-frequency ultrasound and other point-of-care imaging modalities in aesthetic clinical practice. Over the past decade, dermatologic and aesthetic ultrasound has progressed from an occasional adjunct to a recognized component of pre-procedural planning, intra-procedural guidance, and post-procedural evaluation, supported by international guidelines and consensus documents (17–19). Specific applications in current practice include vascular mapping prior to filler injection to mitigate intravascular events, the identification of previously injected products and the characterization of their behavior over time, and the assessment of inflammatory or infectious complications, including granulomas, foreign-body reactions, and abscess-like collections (20–22).

The growing role of these techniques is fully consistent with the OSP. Imaging findings—vascular topography, depth and echogenic pattern of injected products, distinguishing features of granulomatous versus nodular deposits, and signs of inflammation—constitute additional inferential channels that update prior expectations about latent anatomical and pathological states, rather than yielding observer-independent measurements. Operator experience, probe frequency, scanning protocol, and reporting conventions all condition the interpretation of any acquired image, in line with the broader emphasis on standardization and operator-dependence in dermatologic ultrasound (17, 18). Even a quantitatively reproducible parameter, such as the depth of a labial artery from the skin surface, becomes clinically meaningful only once embedded in a specific decision structure that weighs anatomical priors, intended procedural plane, and risk thresholds.

Within the OSP framework, the value of bedside imaging in aesthetic practice is therefore not in providing a privileged ground truth independent of the observer, but in improving the inferential update that informs both the procedural decision and the subsequent evaluation. Pre-injection vascular mapping can be understood as a procedure-level inferential refinement that reduces uncertainty about local anatomy at the moment of treatment. Detection of previously placed material allows the planner to revise prior assumptions regarding what is being treated and at which tissue plane. The differentiation between filler nodules, fibrotic tissue, oedema, granulomas, and abscess-like collections constitutes a complication-level inferential update relevant to the management of adverse events. In each case, ultrasound does not bypass the inferential structure of aesthetic clinical evaluation; it enriches it.

This recognition has direct implications for study design, reporting, and comparison. When imaging adjuncts are used, study reports should make explicit the equipment, frequency range, operator profile, scanning protocol, and the inferential question that the imaging is intended to address—vascular safety, identification of previous products, complication characterization, or longitudinal follow-up—as recommended by recent consensus documents (18, 19). From the OSP perspective, this transparency is not an additional methodological constraint but a faithful reflection of the inferential nature of the information ultrasound provides. Conflating ultrasound findings across heterogeneous protocols, devices, or operators can otherwise generate the same kind of category errors discussed previously for composite endpoints, with the appearance of objective measurement masking a structurally inferential process.

### 8.4 Interpretation of outcomes and apparent discrepancies

Many apparent discrepancies in aesthetic clinical research, such as divergent outcomes across studies, limited reproducibility, or disagreement between patient-reported and clinician-rated measures, are commonly attributed to subjectivity, bias, or measurement error.

The OSP provides an alternative interpretation. When outcomes are understood as inferential updates rather than as direct measurements, discrepancies naturally arise from differences in prior expectations, observer models, and contextual assumptions. Disagreement between observers has been extensively documented in the evaluation of clinical outcomes and is not fully reducible to measurement error, even when standardized instruments are used (23).

Such differences do not imply that one result is correct and another incorrect; rather, they indicate that different inferential questions are being answered (9), as described in Box 1.

Box 1 **Same observations, different inferential levels: why apparent disagreements are not contradictions.**

Consider a patient evaluated at the beginning of the study and at 12 weeks following an intervention. post-treatment documentation includes standardized photographs, a clinician-rated improvement scale, and a PRO. The photographs are judged by the treating physician as showing a clear improvement in contour and proportion, and the clinician assigns a high improvement rating. In parallel, the patient reports low satisfaction, citing a sense of “unnaturalness” and a mismatch with their expected identity-related appearance. A third-party rater, viewing the same images without clinical context, assigns a moderate improvement rating and considers the result natural.

Under a procedure-centered interpretation, these results may be treated as inconsistent measurements of the same outcome. Under the OSP, the apparent discrepancy is expected and structurally interpretable. The clinician rating reflects primarily a decision-level judgment informed by clinical priors and an implicit model of acceptable change. The patient-reported score reflects a subjective-level update shaped by individual expectations and identity-related priors. The third-party assessment reflects a perceptual-level judgment conditioned on different contextual information and priors.

All three evaluations are inferential updates about latent states conditioned on different observer models and different inferential levels. The disagreement is therefore not evidence of measurement failure but evidence that distinct inferential questions are being answered. Meaningful comparison requires an explicit specification of the observer's perspective, context, and the intended inferential level of each instrument.

From this perspective, variability is not a defect that should be eliminated, but an information to be interpreted. Recognizing discrepancies as inferential rather than purely methodological allows aesthetic clinical research to move beyond binary notions of success and failure toward a more nuanced and transparent interpretation.

## 9 Scope, limits, and non-claims of the Open State Principle

The Open State Principle is intentionally limited in scope. Its purpose is not to replace existing methodologies, redefine clinical standards, or prescribe aesthetic practices, but rather to clarify the inferential structure that underlies the aesthetic clinical evaluation. This clarification concerns the conditions under which aesthetic results can be meaningfully defined, not the legitimacy of existing clinical practices or instruments. Explicit articulation of these limits is essential to avoid misinterpretation of the intent and implications of the principle, particularly in domains where conceptual clarification may be mistaken for normative guidance (9). This clarification concerns the conditions under which aesthetic outcomes can be coherently defined for interpretation and comparison; it does not deny the pragmatic legitimacy of locally validated instruments used for context-bound decision support.

First, the OSP does not claim to define what constitutes a desirable or optimal aesthetic outcome. Questions of aesthetic value, preference, or cultural normativity are beyond its scope. The principle addresses how aesthetic outcomes are inferred and interpreted, not which outcomes should be pursued. This distinction parallels established separations in outcome research between descriptive or interpretive frameworks and value-based judgments (10).

Second, the OSP does not provide clinical guidance, treatment algorithms, or decision rules. Although personalization is characterized as decision-making under uncertainty, it does not specify how decisions should be made, which interventions should be selected, or how risks and benefits should be weighted. Such determinations remain the responsibility of clinicians, patients, and regulatory bodies, consistent with the predominant models of shared decision-making and clinical governance (12).

Third, the OSP does not invalidate existing outcome measures, validated scales, or patient-reported instruments. It neither challenges their legitimacy nor proposes alternatives. Instead, it situates these instruments within an inferential framework that clarifies their meaning, scope, and limitations without altering their practical use. This positioning is consistent with methodological analyzes that emphasize that interpretive frameworks can coexist with established instruments without compromising their validity (4).

Fourth, the OSP does not assert that aesthetic clinical systems must be modeled using a specific mathematical formalism. Although the principle is compatible with Bayesian and decision-theoretic structures, it does not mandate their implementation. The absence of a complete formal model in the present work reflects a deliberate acknowledgment of the current heterogeneity of aesthetic clinical practice and the risks associated with premature formal closure (14).

Finally, the OSP should be understood as a principle of second order. It operates at a level above empirical findings, clinical techniques, and evaluative instruments. Its role is to impose coherence constraints on how aesthetic clinical phenomena are described and related, rather than to generate direct predictions or testable hypotheses. In this respect, it functions analogously to other foundational principles that constrain interpretation without competing with empirical models (8).

### 9.1 Operational meaning of incoherence

Within the OSP framework, a “violation” is not an empirical refutation but a category error. Examples include treating a decision-level composite score as an observer-independent biological quantity; comparing judgments of naturalness across studies without specifying observer model alignment; or interpreting regeneration as an observable endpoint in the absence of an explicit causal model. Such violations can be detected through the analysis of study definitions, endpoint construction, and measurement mappings, independently of the size of the effect or statistical significance (7).

By clearly delineating what the OSP does and does not claim, this section aims to prevent normative, ethical, or prescriptive interpretations that would exceed the intended scope of the principle. The value of the OSP lies in its capacity to unify, clarify, and constrain discourse across aesthetic clinical systems, not in offering definitive answers to aesthetic or clinical questions.

## 10 Conclusion

This work introduced the OSP as a foundational framework for understanding aesthetic clinical evaluation across aesthetic surgery, non-surgical aesthetic medicine, dermatology, and related domains. The OSP reframes aesthetic outcomes, naturalness, treatment response, and personalization as inferential constructs operating in latent states under irreducible uncertainty, rather than as directly observable properties of treated anatomy. This reframing aligns aesthetic clinical evaluation with established accounts of outcome evaluation in medicine, where evaluative judgments are understood as interpretations conditioned on context, observation, and expectation rather than as direct measurements.

By redefining the analysis unit as the esthetic clinical system, the OSP provides a unified conceptual structure capable of accommodating the full range of aesthetic interventions. This system-level perspective clarifies why heterogeneous procedures can be evaluated using similar outcome concepts while also explaining persistent difficulties in comparison, replication, and interpretation between studies. Hence, apparent inconsistencies are understood as consequences of differing inferential contexts and observer models, rather than as intrinsic failures of methodology or instrumentation.

The principal contribution of the OSP lies not in proposing new metrics or replacing existing instruments, but in offering epistemic clarification. Validated scales, patient-reported outcomes, and regulatory frameworks remain fully legitimate within this perspective, but are interpreted as local inferential tools rather than as direct measures of aesthetic states. From this point of view, discrepancies between observers, instruments, or contexts are reinterpreted as differences in inference, rather than as evidence of measurement error or methodological inadequacy.

As a principle of second order, the OSP does not seek to resolve aesthetic questions, prescribe clinical practice, or impose formal models. Its role is to impose minimal coherence constraints on the way aesthetic phenomena are described, evaluated, and related. By doing so, it establishes a conceptual foundation on which future methodological, empirical, and mathematical developments in aesthetic clinical research may be coherently built, without constraining the diversity of clinical practices or evaluation instruments currently in use.

This positioning is consistent with the long-standing recognition that medical practice combines scientific evidence with interpretive judgment exercised under uncertainty (24). The OSP does not adjudicate between the “art” and “science” of aesthetic medicine; rather, it articulates the minimal rules of engagement under which heterogeneous instruments, observers, and contexts can communicate coherently about aesthetic outcomes. Within these constraints, the legitimate diversity of aesthetic judgements—across cultures, observers, and clinical traditions—is preserved, while the structural requirements for meaningful comparison and interpretation are made explicit.

## Data Availability

The original contributions presented in the study are included in the article/Supplementary material, further inquiries can be directed to the corresponding author.

## References

[1] Gutowski KS Chwa ES Weissman JP Garg SP Simmons CJ Brandt KE . Practice profile of practicing plastic surgeons: a 20-year review of plastic surgery statistics. Plast Reconstruct Surg Glob Open. (2023) 11:e5486. doi: 10.1097/GOX.0000000000005486 38145152 PMC10745238

[2] Jia X Feng Y. Energy-based skin rejuvenation: a review of mechanisms and thermal effects. J Cosmet Dermatol. (2025) 24:e16657. doi: 10.1111/jocd.16657 39485034 PMC11837243

[3] White C Brahs A Dorton D Witfill K. Platelet-rich plasma: a comprehensive review of emerging applications in medical and aesthetic dermatology. J Clin Aesthet Dermatol. (2021) 14:44–57. 34980960 PMC8675348

[4] Pusic AL Klassen AF Scott AM Klok JR Cordeiro PG Cano SJ. Patient-reported outcome measures in plastic surgery: use and interpretation in evidence-based medicine. Plast Reconstr Surg. (2011) 127:1361–7. doi: 10.1097/PRS.0b013e3182063276 21088640

[5] Rhee JS McMullin BT. Outcome measures in facial plastic surgery: patient-reported and clinical efficacy measures. Arch Facial Plast Surg. (2008) 10:194–207. doi: 10.1001/archfaci.10.3.194 18490547

[6] Pusic AL Klassen AF Scott AM Cano SJ. Development and psychometric evaluation of the FACE-Q satisfaction with appearance scale: a new patient-reported outcome instrument for facial aesthetics patients. Clin Plast Surg. (2013) 40:249–60. doi: 10.1016/j.cps.2012.12.001 23506765

[7] McKenna SP Heaney A. Composite outcome measurement in clinical research: the triumph of illusion over reality? J Med Econ. (2020) 23:1196–204. doi: 10.1080/13696998.2020.1797755 32673124

[8] Friston K. The free-energy principle: a unified brain theory? Nat Rev Neurosci. (2010) 11:127–38. doi: 10.1038/nrn2787 20068583

[9] Gelman A Hennig C. Beyond subjective and objective in statistics. J R Stat Soc: Ser A (Stat Soc). (2017) 180:967–1033. doi: 10.1111/rssa.12276

[10] Guyatt GH Feeny DH Patrick DL. Measuring Health-Related Quality of Life. Ann Intern Med. (1993) 118:622–9. doi: 10.7326/0003-4819-118-8-199304150-00009 8452328

[11] U S Food and Drug Administration. Guidance for Industry: Patient-Reported Outcome Measures: Use in Medical Product Development to Support Labeling Claims. FDA Guidance Document (2009). Silver Spring, MD: U.S. Food and Drug Administration.

[12] Patrick DL Burke LB Gwaltney CJ Leidy NK Martin ML Molsen E . Content validity–establishing and reporting the evidence in newly developed patient-reported outcomes (PRO) instruments for medical product evaluation: ISPOR PRO Good Research Practices Task Force report: part 2–assessing respondent understanding. Value Health. (2011) 14:978–88. doi: 10.1016/j.jval.2011.06.013 22152166

[13] Prinsen CAC Mokkink LB Bouter LM Alonso J Patrick DL de Vet HCW . COSMIN guideline for systematic reviews of patient-reported outcome measures. Qual Life Res. (2018) 27:1147–57. doi: 10.1007/s11136-018-1798-3 29435801 PMC5891568

[14] Jaynes ET. Probability Theory: The Logic of Science. Cambridge, MA: Cambridge University Press (2003). doi: 10.1017/CBO9780511790423

[15] Bernardo JM Smith AFM. Bayesian Theory. Chichester: Wiley (1994). doi: 10.1002/9780470316870

[16] Boutron I Altman DG Moher D Schulz KF Ravaud P. CONSORT statement for randomized trials of nonpharmacologic treatments: a 2017 update and a CONSORT extension for nonpharmacologic trial designs. Ann Intern Med. (2017) 167:40–7. doi: 10.7326/M17-0046 28630973

[17] Wortsman X Alfageme F Roustan G Arias-Santiago S Martorell A Catalano O . Guidelines for performing dermatologic ultrasound examinations by the DERMUS group. J Ultrasound Med. (2016) 35:577–80. doi: 10.7863/ultra.15.06046 26887446

[18] Chammas MC Sigrist R Alfageme F Gonzalez C Cavallieri F Desyatnikova S . WFUMB position paper: consensus on best practice in aesthetic dermatologic ultrasound. Ultrasound Med Biol. (2025) 51:2173–93. doi: 10.1016/j.ultrasmedbio.2025.07.003 40866164

[19] Velthuis PJ Alfageme F Cartier H Schelke L Wortsman X. The use of ultrasound imaging in aesthetic injectables: a modified Delphi consensus. J Plast Reconstruct Aesthet Surg. (2025) 104:33–7. doi: 10.1016/j.bjps.2025.03.001 40101353

[20] Almushayt SJ Alharbi AB Almushayt SJ Algarni HM Almushayt MS. The role of ultrasound in facial hyaluronic acid dermal filler injections–a review article. Clin Imaging. (2025) 120:110424. doi: 10.1016/j.clinimag.2025.110413 39884168

[21] Cavallieri FA Balassiano LKA Munhoz G Tembra MF Wortsman X. Ultrasound in aesthetics: filler and non-filler applications. Semin Ultrasound CT MR. (2024) 45:251–63. doi: 10.1053/j.sult.2023.11.005 38072289

[22] Beiu C Popa LG Bălăceanu-Gurău B Iliescu CA Racoviţă A Popescu MN . Personalization of minimally-invasive aesthetic procedures with the use of ultrasound compared to alternative imaging modalities. Diagnostics. (2023) 13:3512. doi: 10.3390/diagnostics13233512 38066753 PMC10705986

[23] de Vet HCW Terwee CB Knol DL Bouter LM. When to use agreement versus reliability measures. J Clin Epidemiol. (2006) 59:1033–9. doi: 10.1016/j.jclinepi.2005.10.015 16980142

[24] Montgomery K. How Doctors Think: Clinical Judgment and the Practice of Medicine. New York, NY: Oxford University Press (2006). doi: 10.1093/oso/9780195187120.001.0001

