# Supplementary material for: The Open State Principle: a second-order framework for outcome interpretation and decision-making in aesthetic clinical systems
Source: Front Med (Lausanne). 2026 Jun 23;13:1783056. doi: 10.3389/fmed.2026.1783056 (PMC13338874; doi:10.3389/fmed.2026.1783056)
Supplement: Supplementary file 1 [file Data_Sheet_1.pdf]

Supplementary Material S1

MINIMAL FORMAL SKELETON OF THE OPEN STATE PRINCIPLE

S1.1 Scope and purpose of the formal skeleton

This supplementary material provides a minimal formal skeleton for the Open State Principle (OSP). Its purpose is not to introduce an operational or fully specified mathematical model, but to demonstrate that the conceptual structure articulated in the main text admits a coherent inferential formalization.

Accordingly, the notation introduced here is deliberately abstract and underspecified. No assumptions are made about the dimensionality of the state space, the form of probability distributions, or the specific nature of observational mappings. This skeleton is intended solely to clarify the logical relations between states, observations, observers, and decisions and to support future formal developments.

S1.2 Latent aesthetic states

Let $x \in \mathcal{X}$ denote a latent aesthetic state, representing the underlying biological–structural configuration relevant to aesthetic evaluation. The state x is not directly observable and does not correspond to any single measurable quantity.

The space $\mathcal{X}$ is not specified and may include anatomical, physiological, and structural dimensions, depending on the clinical context.

S1.3 Observations and observer dependence

Let $y \in \mathcal{Y}$ denote an observation, where observations may include clinical examination, imaging, photographic data, or perceptual impressions.

Crucially, observations are conditioned not only on the latent state x, but also on an observer $o \in \mathcal{O}$, which encodes perceptual, cognitive, and contextual factors.

This relation can be expressed generically as:

$$y \sim p(y \mid x, o)$$

No assumption is made that different observers share the same observational model. Observer dependence is therefore explicitly represented, rather than treated as noise.

S1.4 Prior expectations and baseline states

Let $p(x \mid o)$ denote the prior belief of observer o about the latent aesthetic state. This prior implicitly encodes baseline expectations, normative standards, and contextual assumptions.

Baseline assessments in aesthetic clinical practice correspond to the explicit or implicit specification of such priors.

S1.5 Outcomes as inferential updates

Given an observation y, the observer updates beliefs about the latent state according to Bayes' rule:

$$p(x \mid y, o) \propto p(y \mid x, o)\, p(x \mid o)$$

Within the OSP framework, an aesthetic outcome is identified with this inferential update, rather than with the posterior state itself or with the observation alone.

Thus, outcomes are relational quantities, defined relative to both prior expectations and observer models.

S1.6 Decision-making under uncertainty

Let $a \in \mathcal{A}$ denote a therapeutic or evaluative decision, such as treatment selection, classification of response, or determination of success.

Decisions are based on beliefs about latent states and may be represented as:

$$a = \arg\min_{a \in \mathcal{A}} \mathbb{E}_{p(x|y,o)} \left[C(x, a) \right]$$

where $C(x, a)$ denotes a cost or loss function that encodings clinical, aesthetic, or patient-centered considerations.

The specific form of C is not specified, reflecting the diversity of decision criteria in esthetic clinical contexts.

S1.7 Levels of description and aggregation

Variables associated with different inferential levels, biological states x, observations y, subjective judgments, and decisions a are not assumed to be directly commensurable.

Any aggregation across levels requires an explicit mapping. In the absence of such a mapping, the composite quantities lack a well-defined inferential interpretation.

S1.8 Relation to the main text

The formal relations outlined above correspond directly to the axioms and derivations presented in the main text. No additional assumptions are introduced. This skeleton is intended as a conceptual bridge between the foundational principles articulated in the article and future domain-specific mathematical implementations.
