# Supplementary material for: The Open State Principle: a second-order framework for outcome interpretation and decision-making in aesthetic clinical systems
Source: Front Med (Lausanne). 2026 Jun 23;13:1783056. doi: 10.3389/fmed.2026.1783056 (PMC13338874; doi:10.3389/fmed.2026.1783056)
Supplement: Supplementary file 2 [file Data_Sheet_2.pdf]

Supplementary Material S2

MATHEMATICAL FORMALIZATION OF THE OPEN STATE PRINCIPLE

S2.1 Purpose and scope

This supplementary material provides a minimal mathematical articulation of the inferential structure underlying the Open State Principle (OSP). Its purpose is illustrative rather than operational. No claims are made about model completeness, identifiability, or empirical adequacy.

S2.2 Latent aesthetic states

Let $x \in \mathcal{X}$ denote a latent aesthetic state. The state space $\mathcal{X}$ may include anatomical, structural, and physiological dimensions, but is not restricted to any particular parameterization.

Latent states are not directly observable and are inferred from the data.

S2.3 Observations and observer dependence

Let $y \in \mathcal{Y}$ denote an observation. Observations may include clinical measurements, imaging data, photographic representations, or perceptual reports.

Observer dependence is explicitly represented by conditioning on an observer variable $o \in \mathcal{O}$:

$$p(y \mid x, o)$$

Different observers are not assumed to share identical observational models.

S2.4 Prior expectations and baseline inference

Each observer is associated with a prior distribution over latent states:

$$p(x \mid o)$$

This prior encodes baseline expectations, contextual norms, and evaluative criteria. Baseline assessments in aesthetic practice correspond to the specification of such priors.

S2.5 Outcome as inferential update

Given an observation y, the posterior belief in latent states is given by:

$$p(x \mid y, o) \propto p(y \mid x, o)\, p(x \mid o)$$

Within the Open State Principle framework, an aesthetic outcome corresponds to the inferential update from prior to posterior, not to the posterior state itself.

S2.6 Decision-making under uncertainty

Let $a \in \mathcal{A}$ denote a therapeutic or evaluative decision. Decisions are selected by minimizing expected loss:

$$a^* = \arg\min_{a \in \mathcal{A}} \mathbb{E}_{p(x|y,o)} \left[C(x, a) \right]$$

The cost function $C(x, a)$ remains unspecified and may vary in clinical, aesthetic, and patient-centered contexts.

S2.7 Relation to the main text

The formal relations presented here correspond directly to the axioms and derivations articulated in the main text. They are not intended to define a complete model, but to demonstrate that the Open State Principle admits a coherent mathematical representation compatible with established inferential frameworks. The relations specified here also imply that, in the absence of explicit indexing to observer models and baseline expectations, aesthetic outcome evaluation does not define a unique inferential mapping, a consequence discussed in the main text.
