# Supplementary material for: The Open State Principle: a second-order framework for outcome interpretation and decision-making in aesthetic clinical systems
Source: Front Med (Lausanne). 2026 Jun 23;13:1783056. doi: 10.3389/fmed.2026.1783056 (PMC13338874; doi:10.3389/fmed.2026.1783056)
Supplement: Supplementary file 3 [file Data_Sheet_3.pdf]

Supplementary Material S3

INFERENTIAL GLOSSARY FOR AESTHETIC CLINICAL SYSTEMS

Latent aesthetic state

A non-observable configuration of biological and structural properties that underlies aesthetic evaluation and is inferred from observations.

Observation

Any clinical, perceptual, instrumental, or reported data item that provides indirect information about a latent aesthetic state.

Observer

An individual or system (e.g., clinician, patient, assessor) whose perceptual and evaluative model mediates the interpretation of observations.

Outcome

An inferential update relative to an expected or baseline state, rather than a post-intervention measurement.

Treatment response

A transformation in belief about a latent aesthetic state conditioned on new observations, not a static post-treatment attribute.

Naturalness

A relational judgment emerging from the interaction between a latent state, an observer model, and contextual expectations.

Personalization

Decision-making under uncertainty that reflects the selection of an action based on expected consequences rather than deterministic optimization.

Inferential level

A distinct level of description (biological, perceptual, subjective, decision-level) at which variables operate and acquire meaning.

CONCEPTUAL MAP OF INFERENTIAL LEVELS, INSTRUMENTS, AND INTERPRETATIONS

Aesthetic clinical evaluation can be represented as a mapping across inferential levels:

- **Biological–structural level**
  Latent states are inferred from anatomical changes, tissue properties, and physiological processes.

- **Perceptual level**
  Observable features such as contour, symmetry, texture, and proportion as interpreted by an observer.
- **Subjective level**
  Individual judgments, satisfaction, expectations, and patient-reported outcomes.
- **Decision level**
  Clinical and regulatory determinations, including classification of the success, response, or adequacy of treatment.

Instruments commonly used in aesthetic clinical research operate at different levels or aggregate across levels. Their outputs acquire meaning only when interpreted in relation to the inferential level at which they function. Explicit recognition of this structure clarifies the scope, limitations, and appropriate use of aesthetic outcome measures.
