# Supplementary material for: The Open State Principle: a second-order framework for outcome interpretation and decision-making in aesthetic clinical systems
Source: Front Med (Lausanne). 2026 Jun 23;13:1783056. doi: 10.3389/fmed.2026.1783056 (PMC13338874; doi:10.3389/fmed.2026.1783056)
Supplement: Supplementary file 4 [file Data_Sheet_4.pdf]

Supplementary Material S4

FORMAL RESULTS FOR THE OPEN STATE PRINCIPLE

S4.1 Formal definition of an aesthetic clinical system

An aesthetic clinical system is defined as a tuple

$$\mathcal{S} = (\mathcal{X}, \mathcal{Y}, \mathcal{O}, p(y \mid x, o), p(x \mid o), \mathcal{A}, C),$$

where $\mathcal{X}$ denotes a space of latent aesthetic states, $\mathcal{Y}$ a space of observations, and $\mathcal{O}$ a set of observers. The conditional distribution $p(y \mid x, o)$ represents observer-dependent observation models, while $p(x \mid o)$ encodes observer-indexed baseline expectations or prior beliefs over latent states. The set $\mathcal{A}$ denotes admissible actions or evaluative decisions, and $C : \mathcal{X} \times \mathcal{A} \to \mathbb{R}$ is a cost or loss function encoding clinical, aesthetic, or patient-centered criteria.

No assumptions are made about the dimensionality of $\mathcal{X}$ or $\mathcal{Y}$, the parametric form of the distributions, or the specific nature of the observation and decision processes.

S4.2 Outcomes as inferential functionals

Within this framework, an aesthetic outcome is not identified with a post-intervention state or an observed value, but with an inferential functional defined relative to an observer. One general formulation expresses the outcome associated with an observation y for the observer o as a reduction in expected risk.

$$\Delta R_o(y) = \inf_{a \in \mathcal{A}} \mathbb{E}_{p(x|o)}[C(x, a)] - \inf_{a \in \mathcal{A}} \mathbb{E}_{p(x|y,o)}[C(x, a)].$$

This definition captures the result as an improvement in uncertainty-relevant expectations in decision making, without prescribing a specific cost function or decision rule. Alternative formulations, such as divergences between prior and posterior beliefs, may be adopted without altering the inferential structure.

S4.3 Proposition 1: Non-existence of observer-invariant outcome mappings

0.0.0.1 Proposition.

If outcome evaluation depends constitutively on observer-indexed inferential updates over latent states, then there is generally no observer-invariant mapping $g : \mathcal{Y} \to \mathcal{Z}$ that can represent aesthetic outcomes independently of the observer.

0.0.0.2 Proof.

Assume, in contradiction, that such a mapping g exists. By definition, for any observation $y \in \mathcal{Y}$ and any observer $o \in \mathcal{O}$, the value $g(y)$ must coincide with the result inferred by o from y. However, if there exist observers o_1, o_2 such that the corresponding inferential updates $p(x \mid y, o_1)$ and $p(x \mid y, o_2)$ differ, then the resulting outcomes, as defined by $\Delta R_{o_1}(y)$ and $\Delta R_{o_2}(y)$, cannot coincide. This contradicts the assumed observer invariance of g. Hence, such mappings generally do not exist.

S4.4 Proposition 2: Non-identifiability of cross-level composite scores

0.0.0.3 Proposition.

Composite outcome measures aggregating variables defined at distinct inferential levels are non-identifiable unless an explicit mapping between those levels is specified.

0.0.0.4 Proof sketch.

Variables associated with biological states, perceptual features, subjective judgments, and decision criteria inhabit distinct spaces and do not need to admit a canonical mapping. Aggregation without an explicit inter-level transformation defines multiple incompatible composite values consistent with the same observations. Consequently, the composite score lacks a unique inferential interpretation.

S4.5 Proposition 3: Locality of naturalness metrics

0.0.0.5 Proposition.

Measures of naturalness are necessarily local to specific observer models and contextual assumptions and cannot define a global observer-independent ordering of aesthetic states.

0.0.0.6 Proof sketch.

Let naturalness be represented as a function $N(x, o, c)$ of a latent state x, an observer o, and contextual factors c. If observer models or contexts differ, then for two states x_1, x_2 it is possible that $N(x_1, o_1, c_1) > N(x_2, o_1, c_1)$ while $N(x_1, o_2, c_2) < N(x_2, o_2, c_2)$. Hence, there is no total ordering invariant between observers and contexts.

S4.6 Corollary: Ill-posedness of observer-independent aesthetic evaluation

0.0.0.7 Corollary.

In the absence of explicit indexing to observer models, baseline expectations, and inferential level, aesthetic outcome evaluation is ill-posed, in the sense that it does not admit a unique and well-defined solution.

S4.7 Implementation note (non-prescriptive)

The formal structure outlined above does not prescribe specific instruments, cost functions, or decision rules. Existing clinical scales, patient-reported outcomes, and regulatory endpoints can be located within this framework by specifying the observer perspective, baseline expectations, and the inferential level at which they operate. The Open State Principle thus constrains interpretation without dictating implementation.
